# Phagentherapeutika und Arzneimittelrecht: Klassifikation, Herstellung, Inverkehrbringen und Anwendung

**DOI:** 10.1007/s00103-025-04054-0

**Published:** 2025-05-09

**Authors:** Timo Faltus

**Affiliations:** Juristische und Wirtschaftswissenschaftliche Fakultät, Juristischer Bereich, Martin-Luther-Universität Halle-Wittenberg, Universitätsplatz 3–5, 06099 Halle (S.), Deutschland

**Keywords:** Phagenarzneimittel, Rezepturarzneimittel, Personalisierte Medizin, Phagenlysine, Krankenhausapotheke, Phage therapy medicinal products, Extemporaneous preparation, Personalized medicine, Phage lysins, Hospital pharmacy

## Abstract

Nach heutigem Stand wird der überwiegende Teil von Phagentherapeutika individualisiert, also eigens für einen bestimmten Patienten hergestellt. Hierbei findet die Herstellung in der Regel dezentral in den jeweiligen Krankenhausapotheken als Rezepturarzneimittel statt. Damit unterscheidet sich die technische Bereitstellung von Phagenarzneimitteln vom Leitbild des Arzneimittelrechts. Die Sicherung von Qualität, Wirksamkeit und Unbedenklichkeit von Arzneimitteln ist typischerweise an die Bereitstellung von patientenunabhängig, vorab, zentral hergestellten und dezentral angewendeten Arzneimitteln angepasst. Lediglich in bestimmten gesetzlich normierten Ausnahmefällen dürfen Arzneimittel dezentral von Apotheken oder Ärzten hergestellt werden, ohne dass eine Herstellungserlaubnis und ohne dass vor Anwendung eine Zulassung notwendig ist.

Der Übersichtsbeitrag zeigt, warum Phagenarzneimittel aus Wildtypphagen aus rechtlicher Sicht lediglich einfache Funktionsarzneimittel darstellen. Darauf aufbauend wird erläutert, unter welchen rechtlichen Voraussetzungen individualisierte und nichtindividualisierte Phagenarzneimittel hergestellt, in den Verkehr gebracht und angewendet werden können. Zudem wird auf Rechtsfragen in Bezug auf Phagenlysine eingegangen. Weiterhin zeigt der Beitrag die Auswirkungen der arzneimittelrechtlichen Handhabung von Phagentherapeutika auf die Voraussetzungen für deren Erstattung durch die gesetzlichen Krankenkassen. In Anbetracht der technischen Voraussetzungen der Phagentherapie wird erörtert, inwieweit der heutige Rechtsrahmen die Translation von Phagenarzneimitteln in die medizinische Regelversorgung ermöglicht. Schließlich wird eine Übersicht zu geplanten Gesetzesänderungen und deren voraussichtlichen Auswirkungen auf die rechtliche Handhabung von Phagenarzneimitteln gegeben.

## Abstract (en)

Currently, most phage therapeutics are individualized, that is, prepared specifically for a single patient. In this case, the preparation is usually decentralized and takes place in the respective hospital pharmacies as extemporaneous preparations. The technical supply of phage medicinal products thus differs from the guiding principle of pharmaceutical law. The assurance of quality, efficacy, and safety of medicinal products is typically adapted to the supply of medicinal products that are centrally manufactured independently of the patient in advance and administered decentrally. Only in legally defined exceptional cases may medicinal products be prepared decentrally by pharmacies or physicians without the need for a manufacturing authorization or a marketing authorization prior to application.

The article shows why, from a legal perspective, phage medicinal products based on wild-type phages are simple, non-specifically classified medicinal product by virtue of function. Based on this, the article describes the legal requirements under which individualized and non-individualized phage medicinal products can be manufactured, placed on the market and administered. The article also addresses legal issues relating to phage lysins. Furthermore, the article shows the impact of the legal handling of phage pharmaceuticals on the requirements for the reimbursement of phage pharmaceuticals by health insurance providers. In view of the technical requirements of phage therapy, the article discusses the extent to which the current legal framework enables the translation of phage medicinal products into standard medical care. Finally, the article provides an overview of currently planned legislative changes and their likely impact on the legal handling of phage medicinal products.

## Einleitung

Herstellung und Inverkehrbringen phagenbasierter Therapeutika zur Behandlung bakterieller Infektionen werden durch das Arzneimittelrecht, hauptsächlich durch das Arzneimittelgesetz (AMG), die Arzneimittel- und Wirkstoffherstellungsverordnung (AMWHV) und die Apothekenbetriebsordnung (ApBetrO), geregelt. Der überwiegende Teil der Phagentherapeutika wird nach heutigem Stand als individualisierte Therapie entwickelt. Dabei werden die Therapeutika jeweils eigens für einen Patienten dezentral, zumeist in Krankenhausapotheken hergestellt [1–3].

Das Leitbild des Arzneimittelrechts ist aber das zentral, vorab, patientenunabhängig hergestellte und dezentral angewendete Fertigarzneimittel. Auf diesem Leitbild bauen die Vorschriften zur Qualität, Wirksamkeit und Unbedenklichkeit auf, die insbesondere in präklinischen und klinischen Studien sowie in hoheitlich kontrollierten Verfahren zur Herstellungserlaubnis und Zulassung zum Inverkehrbringen belegt werden müssen. Ausnahmsweise lässt das Arzneimittelrecht die ärztliche und apothekenhafte Herstellung von Rezepturarzneimitteln ohne Zulassung und teilweise auch ohne Herstellungserlaubnis zu. Allerdings handelt es sich um Ausnahmen für Arzneimittel, die lediglich in einem geringeren Umfang als Fertigarzneimittel bereitgestellt werden. Wenn die Phagentherapie wie mit der gegenwärtig verfolgten Entwicklung hauptsächlich mit dezentral her- und bereitgestellten Therapeutika durchgeführt würde, würde die Regelversorgung dieser Therapie lediglich auf Ausnahmevorschriften beruhen. Somit stellt sich die Frage, inwieweit das heutige Arzneimittelrecht phagenbasierte Humantherapeutika in Anbetracht deren technischer und medizinischer Eigenarten angemessen reguliert und eine Translation in die medizinische Regelversorgung ermöglicht. Zur Klärung dieser Frage geht der Beitrag auf die rechtliche Klassifikation phagenbasierter Therapeutika ein und erläutert die verschiedenen rechtlichen Anforderungen an die nichtindividualisierte und individualisierte Bereitstellung.

## Klassifikation phagenbasierter Therapeutika

### Arzneimittel und kein Biozid.

Die regulatorische Handhabung phagenbasierter Therapeutika setzt deren rechtliche Einordnung voraus. Hierbei reicht für die eindeutige Einordnung gerade bei neu aufkommenden Therapeutika eine lediglich positive Klassifizierung regelmäßig nicht aus, weil dadurch nicht automatisch alle anderen Klassifizierungen ausgeschlossen sind. Vielmehr ist es zum einen notwendig, die Arzneimittelklassen zu bestimmen, die für phagenbasierte Therapeutika nicht einschlägig sind. Zum anderen ist für den Fall, dass mehrere Einordnungen positiv möglich sind, die Abgrenzung (auch als Demarkation bezeichnet) notwendig, um eine eindeutige Klassifikation zu erreichen.

Therapeutika auf Grundlage von Phagen sind aufgrund ihrer therapeutischen Verwendung am oder im Menschen Arzneimittel gemäß § 2 Abs. 1 AMG und nicht etwa Biozide zur Zerstörung von Schadorganismen nach der EU-Verordnung 528/2012, weil Phagen nicht die vom Biozidrecht nach Art. 3 Abs. 2 lit. a) VO (EU) 528/2012 i. V. m. Art. 3 Nr. 1 VO (EG) 1907/2006 geforderte chemische Stoffeigenschaft erfüllen. Viren sind nicht bloß chemische Stoffe und haben auf molekularer Ebene eine andere Struktur als chemische Elemente und ihre Verbindungen [4, 5].

### Keine stofflichen Medizinprodukte.

Zudem sind Phagenarzneimittel keine (stofflichen) Medizinprodukte, da die EU-Medizinprodukteverordnung gemäß Art. 1 Abs. 6 lit. h) VO (EU) 2017/745 nicht für Produkte gilt, die aus Viren bestehen oder solche enthalten, um die Zweckbestimmung des Produkts zu erreichen oder zu unterstützen [4, 5]. Eine davon zu unterscheidende Frage ist, ob Gegenstände, die für die Applikation der Phagenarzneimittel genutzt werden, Medizinprodukte sind. Darauf aufbauend muss gegebenenfalls bestimmt werden, inwieweit durch die technisch-therapeutische Verbindung eines Phagenarzneimittels mit dem Medizinprodukt ein Kombinationsprodukt mit dann jeweils spezifischen Regulierungsanforderungen entsteht.

### Funktionsarzneimittel.

Phagenarzneimittel sind innerhalb der Arzneimitteldefinition des § 2 Abs. 1 AMG Funktionsarzneimittel gemäß § 2 Abs. 1 Nr. 2 lit. a) AMG, da die Wirkweise in rechtlicher Sicht pharmakologisch ist [4, 5]. Als pharmakologische Wirkung wird eine Wechselwirkung zwischen dem Wirkstoff und den Zellstrukturen am/im Anwender, insbesondere den Rezeptoren, verstanden, wobei diese Interaktion in einer direkten Reaktion resultiert oder die Reaktion eines anderen Agens blockiert [6, 7]. Die Wirkung muss dabei einerseits der Gesundheit zuträglich sein und somit die physiologischen Wirkungen positiv beeinflussen, andererseits muss sich die Wirkung vom Verzehr einer üblichen Menge eines Lebensmittels unterscheiden [8]. Dass diese Wechselwirkung bei Phagen nicht in Bezug auf menschliche Zellen, sondern auf die zu bekämpfenden Bakterien am oder im menschlichen Körper erfolgt, ist rechtlich unerheblich. Der Europäische Gerichtshof (EuGH) hat bereits festgestellt, dass von einer pharmakologischen Wirkung im Rechtssinn auch ausgegangen werden kann, wenn die Wechselwirkung zwischen der infrage stehenden Substanz und einem beliebigen im Körper des Anwenders vorhandenen zellulären Bestandteil wie Bakterien, Viren oder Parasiten gegeben ist, wobei es sich nicht notwendigerweise um eine menschliche Zelle handeln muss [7].

Aufbauend auf der Klassifizierung als Funktionsarzneimittel ist zu klären, ob es sich bei Phagenarzneimitteln um eine speziell geregelte Arzneimittelklasse handelt.

### Keine biologischen Arzneimittel.

Phagenarzneimittel auf Basis von Wildtypphagen sind – auch wenn das aufgrund des natürlichen, biologischen Vorkommens von Phagen zu erwarten wäre – keine biologischen Arzneimittel (Biologicals) nach der Definition in Anhang I, Teil 1, 3.2.1.1, b) S. 3–5 Richtlinie (RL) 2001/83/EG, weil sich Phagenarzneimittel keiner der abschließend als Biologicals definierten Arzneimitteltypen zuordnen lassen^1^. Allenfalls Phagentherapeutika auf Grundlage von gentechnisch veränderten Phagen stellen Biologicals dar [4, 5, 9].

### Keine Blut‑/Gewebezubereitungen, keine xenogenen Arzneimittel.

Phagenarzneimittel sind weder Blutzubereitungen (§ 4 Abs. 2 AMG) noch xenogene Arzneimittel (§ 4 Abs. 21 AMG), noch Gewebezubereitungen (§ 4 Abs. 30 AMG), weil Phagen und auch Phagenarzneimittel weder aus lebenden Zellen bestehen noch aus Blut gewonnene Blut‑, Plasma- oder Serumkonserven, Blutbestandteile oder Zubereitungen aus Blutbestandteilen sind oder diese als Wirkstoffe enthalten bzw. weil Phagen keine Tiere sind [4, 5].

### (K)ein Impfstoff.

Phagenarzneimittel zur Behandlung bakterieller Infektionen sind rechtlich keine Impfstoffe gemäß § 4 Abs. 4 AMG, weil Phagenarzneimittel keine Immunität des menschlichen Immunsystems gegenüber der zu behandelnden bakteriellen Infektion hervorrufen. Die Wirkung der Phagen wird nicht über das Immunsystem vermittelt und vermittelt keine Immunität gegen eine erneut auftretende Infektion, weder mit demselben Bakterium noch gegen ein anderes Bakterium. Im Falle der erneuten Infektion muss eine erneute Phagen- und/oder Antibiotikabehandlung erfolgen, womit die Phagentherapie wie die Antibiotikatherapie gegen bakterielle Infektionen nicht präventiv, sondern kurativ ist [4, 5].

Allerdings könnten sich Phagen mit dem Phagen-Display (Präsentation von Antikörpern auf der Phagenoberfläche) auch präventiv gegen Infektionskrankheiten einsetzen lassen [10, 11] und stellen dann auch aus rechtlicher Sicht Impfstoffe dar. Der Wirkmechanismus ist in diesem Fall über das Immunsystem vermittelt. Während bei der kurativen Nutzung die Phagen die pathogenen Bakterien abtöten, ohne dass das Immunsystem eine Immunität entwickelt, werden beim Phagen-Display Phagen verwendet, um präventiv Immunität gegenüber bestimmten Infektionskrankheiten zu erzeugen. Diese Technik wurde u. a. bei der Entwicklung von Impfstoffen gegen das SARS-CoV-2-Virus untersucht [11]. Die Verwendung von Phagen zur Impfung im Phagen-Display setzt eine genetische Veränderung der Phagen mittels rekombinanter Nukleinsäuren voraus [10]. Dies ändert jedoch nichts an der rechtlichen Einordnung als Impfstoff, da zumindest Impfstoffe gegen Infektionskrankheiten, die eine rekombinante Nukleinsäure enthalten, aufgrund von Art. 2 Abs. 1 lit. a), 1. Tiret VO (EG) Nr. 1394/2007, Anhang I, Teil IV, 2.1, S. 4 RL 2001/83/EG rechtlich Impfstoffe sind und nicht Gentherapeutika (vgl. folgend die Abgrenzung zu Arzneimitteln für neuartige Therapien). Für die Impfverwendung von Phagen sind die Vorschriften für Impfstoffe einschlägig, die aufgrund des hier behandelten Themenschwerpunkts zur Verwendung von Phagen bei bakteriellen Infektionen nicht dargestellt sind.

### Arzneimittel für neuartige Therapien.

Die Vorschriften der Arzneimittel für neuartige Therapien (ATMP, Advanced Therapy Medicinal Products) nach Art. 2 Abs. 1 lit. a) VO (EG) Nr. 1394/2007, § 4 Abs. 9 AMG sind auf Arzneimittel auf Basis von Wildtypphagen nicht anwendbar, weil diese Phagenarzneimittel nicht die für ATMP geforderte zelluläre Eigenschaft haben oder keine rekombinante Nukleinsäure enthalten oder daraus bestehen. Allerdings können bestimmte Arzneimittel auf Grundlage von genetisch veränderten Phagen rechtlich als Gentherapeutika und damit als ATMP gemäß Art. 2 Abs. 1 lit. a), 1. Tiret VO (EG) 1394/2007 i. V. m. Anhang I, Teil IV, 2.1 RL 2001/83/EG gewertet werden, sofern sie eine rekombinante Nukleinsäure im Sinne der Definition des Gentherapeutikums enthalten [12]. Unter Berücksichtigung dieser rechtlich ausdrücklich niedergelegten Anforderungen dürfen sogenannte trainierte Phagen, also Wildtypphagen, die mittels eines genetischen Mutationsverfahrens gewonnen werden und jedenfalls keine rekombinanten Nukleinsäuren enthalten, ebenso wie anderweitig, z. B. mittels der Verfahren der Genomeditierung und damit einschließlich des CRISPR/Cas-Verfahrens, mutierte Phagen nicht als Gentherapeutikum klassifiziert werden [4, 5].

### Einfache Funktionsarzneimittel.

Letztlich sind Therapeutika auf Basis von Wildtypphagen, einschließlich lediglich mutierter Phagen, einfache Funktionsarzneimittel gemäß § 2 Abs. 1 Nr. 2 lit. a) AMG, da die Voraussetzungen der spezifisch geregelten Arzneimittelklassen jeweils nicht erfüllt sind. In diesen einfachen Funktionsarzneimitteln sind die Phagen Wirkstoff gemäß § 4 Abs. 19 AMG, da von den Phagen die gewollte arzneiliche Wirkung, namentlich die Bekämpfung pathogener Bakterien, ausgeht. Unter Berücksichtigung von § 3 Nr. 3 AMG, wonach zu den arzneimittelrechtlichen Stoffen auch Mikroorganismen, einschließlich Viren, zählen, handelt es sich bei Phagen in Bezug auf die Herstellung um erlaubnispflichtige Wirkstoffe i. S. v. § 13 Abs. 3 AMG. Die für die Applikation der Phagen notwendigen zusätzlichen Stoffe, wie z. B. Flüssigkeiten zur Suspensierung der Phagen, sind demnach Hilfsstoffe nach § 4 Abs. 20 AMG. Die Summe aus Wirkstoff(en) und Hilfsstoff(en) ist auch bei phagenbasierten Therapeutika je nach Herstellungsform (vgl. § 4 Abs. 1 AMG) entweder ein Fertigarzneimittel oder ein (Rezeptur‑)Arzneimittel.

## Therapie mit industriell gefertigten Phagenarzneimitteln

Bislang gibt es in der EU – soweit ersichtlich – kein industriell gefertigtes und EU-rechtlich zugelassenes Phagenarzneimittel. In der Slowakei und in Tschechien ist mit Stafal® zwar ein Phagentherapeutikum auf dem Markt [13, 14]. Der Markteintritt erfolgte aber vor dem Beitritt beider Länder zur EU.

Für die patientenunabhängige Vorabherstellung von Fertigarzneimitteln können in technischer und ökonomischer Sicht insbesondere polyvalente Phagen in Phagencocktails verwenden werden [2, 15–17]. Hierfür ist eine Herstellungserlaubnis notwendig, die Herstellung selbst muss unter Bedingungen der Good Manufacturing Practice (GMP) erfolgen, § 13 Abs. 1 AMG, § 1 Abs. 1 Nrn. 1,2, § 3 Abs. 1–2 AMWHV.

Zu beachten ist § 55 Abs. 8 AMG, wonach bei der Herstellung nur Stoffe verwendet werden und nur Darreichungsformen angefertigt werden dürfen, die den anerkannten pharmazeutischen Regeln entsprechen. Da diese Vorschrift auf das Arzneibuch Bezug nimmt, müssen bei der Herstellung die Vorgaben des Arzneibuchs berücksichtigt werden. Im Jahr 2024 wurde im Europäischen Arzneibuch zwar ein allgemeines Kapitel „Phage therapy medicinal products“ zu Aspekten der Herstellung von Phagenarzneimitteln aufgenommen [18], allerdings ist dieses Kapitel lediglich informatorisch.

Für das Inverkehrbringen ist eine Zulassung notwendig. Da Arzneimittel auf Grundlage von Wildtypphagen nicht nach Art. 3 Abs. 1 i. V. m. Anhang I VO (EG) 726/2004 im Katalog der Arzneimittel aufgeführt sind, die obligatorisch durch die EU zentral zugelassen werden müssen, kommt eine fakultative EU-Zulassung in Betracht, sofern die hierfür in Art. 3 Abs. 2 VO (EG) 726/2004 genannten Voraussetzungen erfüllt sind, also z. B., dass nachgewiesen wird, dass das Arzneimittel eine bedeutende Innovation in therapeutischer, wissenschaftlicher oder technischer Hinsicht ist. Der Möglichkeit zur fakultativen EU-Zulassung steht nicht entgegen, nur eine nationale Zulassung in Deutschland nach § 21 AMG anzustreben. Jedenfalls setzt die Zulassung insbesondere die erfolgreiche Testung des Arzneimittels in präklinischen und klinischen Studien voraus (vgl. Art. 6 VO 726/2004, Art. 8 Abs. 3 lit. i) RL 2001/83/EG bzw. §§ 21 Abs. 1 S. 1; 22 Abs. 2 AMG). Im Rahmen der Genehmigung der klinischen Studien ist auch eine ethische Begutachtung der jeweiligen Studie obligatorisch, Art. 4 VO (EU) Nr. 536/2014.^2^

Sollte es sich aufgrund der Verwendung von gentechnisch veränderten Phagen um ATMP handeln, ist nach Art. 3 Abs. 1 i. V. m. Anhang I Nr. 1a VO (EG) 726/2004 grundsätzlich eine EU-Zulassung einschlägig. Sofern die Voraussetzungen der Krankenhausausnahme nach Art. 3 Nr. 7 RL 2001/83/EG, § 4b AMG erfüllt sind, können entsprechende ATMP lediglich national genehmigt werden. Hierzu muss es sich u. a. um Arzneimittel handeln, die auf individuelle ärztliche Verschreibung eigens für einen einzelnen Patienten angefertigt werden.

## Individuelle Therapie

### Versorgungsvarianten.

Die individuelle Phagentherapie ist dadurch gekennzeichnet, dass das Phagenarzneimittel je nach dem zu bekämpfenden Bakterium spezifisch auf Abruf hergestellt wird [2, 17, 19]. Die Herstellung des Arzneimittels erfolgt entweder vom Arzt und/oder auf ärztliche Verschreibung von einer (Krankenhaus‑)Apotheke. Bei dieser Herstellung können Ärzte bzw. die Apotheken die jeweils benötigten Phagen technisch prinzipiell selbst im für die Therapie benötigten Umfang vermehren, wozu eine eigene Phagenbank notwendig ist. Da die Unterhaltung einer Phagenbank und die Phagenvermehrung allerdings Tätigkeiten mit einem Spezialisierungs- und Zeitaufwand sind, der nicht nebenbei und lediglich „ab und zu“ erfolgen kann, werden diese Tätigkeiten typischerweise von darauf spezialisierten Einrichtungen übernommen. Diese Einrichtungen beliefern Ärzte bzw. Apotheken. Die verschiedenen Konstellationen werden rechtlich unterschiedlich behandelt.

Alle individualisierten Varianten werden arzneimittelrechtlich durch nationales Recht reguliert. Die europarechtlichen Vorgaben der Arzneimittel-RL 2001/83/EG und Zulassungsverordnung 726/2004 sind nicht einschlägig, denn Arzneimittel aus ärztlicher Herstellung gelten bei Anwendung durch den Arzt im Rechtssinn nicht als „in den Verkehr gebracht“. Das Inverkehrbringen ist jedoch Voraussetzung für die Anwendbarkeit dieser europarechtlichen Vorschriften, Art. 2 Abs. 1 RL 2001/83/EG bzw. Art. 1, 3 VO (EG) 726/2004.

Selbst wenn in der Variante der Apothekenherstellung durch die Übergabe des Arzneimittels an den Arzt ein Inverkehrbringen vorliegt, ist diese Variante durch Art. 3 Nr. 1 RL 2001/83/EG (individuell auf ärztlichen Abruf in Apotheken hergestellte Arzneimittel, Formula magistralis) vom Anwendungsbereich der Richtlinie ausgeschlossen. Die Zulassungsverordnung 726/2004 setzt ihrerseits die Anwendung der RL 2001/83/EG voraus.

### Herstellung in der Krankenhausapotheke.

Bei Verwendung von Wildtypphagen ist die Herstellung nach ärztlicher Verschreibung in der Krankenhausapotheke nach § 13 Abs. 2 Nrn. 1‑2 AMG erlaubnisfrei, die Herstellung richtet sich nach der Apothekenbetriebsordnung. Das Gleiche gilt aufgrund von § 20a AMG für die Wirkstoffherstellung, also für die Vermehrung der Phagen. Bezieht die Apotheke die Phagen als Wirkstoff von einem apothekenexternen Anbieter, ist § 11 Abs. 2 S. 2 ApBetrO zu beachten, wonach in der Apotheke geprüft werden soll, ob diese Wirkstoffe GMP-konform hergestellt worden sind. Inwieweit von dieser Sollvorschrift in begründeten Fällen, z. B. weil es tatsächlich keine GMP-konform hergestellten Phagen gibt, abgewichen werden kann, ist im Einzelfall zu klären [20]. In jedem Fall ist der Apothekenleiter für die ordnungsgemäße Qualität der Ausgangsstoffe nach § 11 Abs. 3 ApBetrO verantwortlich. Zudem müssen auch hier wegen § 55 Abs. 8 AMG die Vorgaben aus dem Arzneibuch eingehalten werden.

Bei der Herstellung von parenteral zu applizierenden Phagentherapeutika ist § 35 Abs. 3, Abs. 4 ApBetrO zu berücksichtigen, sodass ein Reinraum mit einem Luftreinheitsgrad entsprechend den jeweiligen Vorgaben des EU-GMP-Leitfadens vorgeschrieben ist.

Auch wenn die Aushändigung von der Krankenhausapotheke an den Arzt eine Abgabe und damit ein Inverkehrbringen i. S. v. § 4 Abs. 17 AMG darstellt, ist in Bezug auf Phagenarzneimittel auf Grundlage von Wildtypphagen keine Zulassung notwendig. Das Zulassungserfordernis ist an die rechtliche Eigenschaft des Fertigarzneimittels gemäß § 4 Abs. 1 S. 1 AMG gebunden. Die in (Krankenhaus‑)Apotheken auf Abruf hergestellten Arzneimittel sind jedoch gemäß § 4 Abs. 1 S. 1 AMG keine Fertigarzneimittel sondern (Rezeptur‑)Arzneimittel.

Rechtlich anders werden ATMP-Phagenarzneimittel behandelt, für deren Herstellung auch in der Apotheke nach § 13 Abs. 2 Nrn. 1–2, Abs. 2a S. 1 AMG, § 3 Abs. 1–2 AMWHV eine Herstellungserlaubnis unter GMP-Bedingungen vorgeschrieben ist. Zudem wird die Abgabe dieser auf Abruf individualisiert hergestellten Phagenarzneimittel aus der Apotheke z. B. an den behandelnden Arzt auch als eine genehmigungspflichtige Abgabe nach §§ 4 Abs. 17, 4b Abs. 1,3 AMG behandelt. Damit sind vor Abgabe grundsätzlich präklinische und auch klinische Studien notwendig, ausnahmsweise kommen stattdessen (teilweise) z. B. Daten aus Veröffentlichungen und nachträgliche Bewertungen der klinischen Ergebnisse in Betracht, vgl. § 4b Abs. 3 & 5, § 21a Abs. 2 & 8 AMG.

### Phagenvermehrung und Abgabe durch apothekenexterne Phagenbank.

Die Lagerung und Vermehrung von arzneilich genutzten Phagen in einer apothekenexternen Phagenbank sind wegen fehlender Ausnahmen nach § 13 Abs. 1 AMG, § 3 Abs. 1–2 AMWHV erlaubnis- und GMP-pflichtig. Die Abgabe der Phagen als Wirkstoff an Dritte bedarf keiner eigenständigen Zulassung.

### Ärztliche Herstellung.

Die Herstellung und Anwendung patientenindividueller Phagenarzneimittel sind Ärzten nicht per se verboten. Allerdings darf der Arzt bei Wildtypphagen nur das Arzneimittel nach § 13 Abs. 2b S. 1 AMG erlaubnisfrei herstellen. Die Vermehrung der Phagen als Wirkstoffherstellung ist für den Arzt unter Einhaltung der GMP erlaubnispflichtig, weil es sich bei Phagen um erlaubnispflichtige Wirkstoffe nach § 13 Abs. 3 AMG, § 3 Abs. 1–2 AMWHV handelt [21]. Zudem ist zu klären, inwieweit auch die ärztliche Arzneimittelherstellung durch § 55 Abs. 8 AMG das Arzneibuch berücksichtigen muss.^3^ Für den Fall von ATMP-Phagenarzneimitteln ist der gesamte Herstellungsprozess nach § 13 Abs. 2b S. 2 Nr. 1 AMG, § 3 Abs. 1–2 AMWHV erlaubnis- und GMP-pflichtig.

Für die Anwendung der ärztlich hergestellten Phagenarzneimittel durch den herstellenden Arzt ist keine Zulassung bei Wildtypphagen bzw. keine Genehmigung bei ATMP-Phagen notwendig, da in rechtlicher Sicht kein Inverkehrbringen bzw. keine Abgabe vorliegt.

### Einzeleinfuhr.

Eine weitere Möglichkeit der individuellen Versorgung mit Phagenarzneimitteln besteht durch den arzneimittelrechtlich regulierten Einzelimport. Insbesondere im Nicht-EU/EWR-Ausland sind Fertigarzneimittel auf Phagenbasis im Verkehr. Unter den Voraussetzungen des § 73 Abs. 3 AMG können diese Arzneimittel individuell in Deutschland bereitgestellt werden. Dazu muss u. a. eine ärztliche Verschreibung vorliegen, muss das Arzneimittel im betreffenden Land rechtmäßig in Verkehr sein und es darf in Deutschland kein Arzneimittel mit vergleichbarem Wirkstoff zur Verfügung stehen. Die Vorgabe zum fehlenden Wirkstoff ist typischerweise erfüllt, da in Deutschland bislang keine Phagenarzneimittel regelhaft verfügbar sind. Liegen die Voraussetzungen insgesamt vor, ist der Bezug nur durch Apotheken zulässig. Weitergehende Dokumentationsvorschriften finden sich in § 18, 22 ApBetrO.

## Phagenlysine

Phagenlysine^4^ tragen zur Zerstörung der Zellwand des Bakterienwirts bei [22, 23]. Im Ergebnis wird dadurch wie bei Verwendung von „ganzen“ Phagen das Bakterium abgetötet. Die arzneimittelrechtliche Handhabung von (Phagen‑)Lysinen ist im Vergleich zu Phagen „als Ganzes“ bislang aber widersprüchlich.

Bislang wurde z. B. eine Creme auf Grundlage eines Phagenlysins, hier mit dem Wirkstoff „Endobioma“, zuweilen als „Staphefekt SA.100“ bezeichnet, gegen Entzündungen verursachende Bakterien rechtlich als Medizinprodukt klassifiziert [24–27]. Diese Klassifikation setzt voraus, dass die Wirkweise der Lysine weder pharmakologisch, immunologisch noch metabolisch wie von § 2 Abs. 1 AMG, Art. 2 Nr. 1 VO (EU) 2017/745 gefordert ist. Stattdessen müsste die Wirkweise physikochemisch und/oder physikalisch sein. Ein anderes auf dem Wirkstoff „Endolysin HY-133“ aufbauendes Therapeutikum, das gegenwärtig in klinischen Studien gegen *Staphylococcus aureus* geprüft wird, wurde hingegen als Arzneimittel klassifiziert [28, 29]. Zudem wurde die therapeutische Anwendung von Chlorhexidin (chemisches Molekül), das unspezifisch Bakterien ebenfalls durch strukturelle Schädigung der bakteriellen Zelloberfläche abtötet [30–32], bisher als pharmakologisch angesehen. Dementsprechend wurden bakterizide Chlorhexidintherapeutika als Arzneimittel klassifiziert [7, 33]. Inwieweit diese bislang nicht einheitlichen Klassifizierungen von Therapeutika, die die bakterielle Zelloberfläche strukturell schädigen, trotz vergleichbarer Wirkmechanismen bestehen können, muss der rechtliche Diskurs klären.

## Arzneimittelrechtliche Aspekte der Kostenerstattung

Die Regelung der Kostenerstattung für (phagenbasierte) Arzneimittel durch die gesetzliche Krankenversicherung ist Materie des Sozialrechts. Allerdings hat die arzneimittelrechtliche Handhabung Einfluss auf die sozialrechtliche Verordnungsfähigkeit in der gesetzlichen Krankenkasse.

In der gesetzlichen Krankenversicherung haben Versicherte nach § 27 Abs. 1 Nr. 1, 3 SGB V u. a. Anspruch auf notwendige Arzneimittel. Der Anspruch erstreckt sich nach § 12 SGB V grundsätzlich nur auf wirtschaftliche Leistungen. Zum anderen müssen gemäß § 2 Abs. 1 S. 3 SGB V Qualität und Wirksamkeit der Leistungen dem allgemein anerkannten Stand der medizinischen Erkenntnisse entsprechen. Qualität und Wirksamkeit werden insbesondere durch präklinische und klinische Studien sowie einen hoheitlich kontrollierten Zulassungsprozess, in dem die Studienergebnisse auf ihren Aussagegehalt geprüft werden, nachgewiesen. Bei zulassungspflichtigen Fertigarzneimitteln stellt die Zulässigkeit für die Krankenkassen ein Mindestmaß für Wirksamkeit, Zweckmäßigkeit und Notwendigkeit des betreffenden Arzneimittels dar, mit dem die Krankenkassen über ein Kriterium bei der Entscheidung über die Verordnungsfähigkeit verfügen [34]. Dieses Konzept ist bei Rezepturarzneimitteln nicht anwendbar, da sich die arzneimittelrechtliche Verkehrsfähigkeit der Rezepturarzneimittel allein durch die arzneimittel- oder apothekenrechtliche Herstellungserlaubnis ergibt; eine Zulassung mit zugrunde liegenden Studien ist nicht notwendig. Für die nach dem Arzneimittelrecht zulassungsfreien Arzneimittel, und damit für die Mehrheit der heute in Entwicklung befindlichen Phagenarzneimittel, stellt sich damit die Frage, wie die sozialrechtlich geforderte Einhaltung von Qualität und Wirksamkeit bzw. die Sicherstellung der Wirtschaftlichkeit für die Regelversorgung hergeleitet werden sollen. Jedenfalls ist der Nachweis der Wirksamkeit und Unbedenklichkeit für die Verordnungsfähigkeit eines Arzneimittels zulasten der gesetzlichen Krankenkassen unverzichtbar, um die Gesundheit der Patienten und die Beiträge der Versicherten zu schützen [34].

Diese offene Erstattungsfrage führt nicht zu einem absoluten, durch das Sozialrecht bedingten Anwendungsverbot wie bei bedenklichen Arzneimitteln i. S. v. § 5 AMG, da nur die Anwendung innerhalb, nicht aber außerhalb des sozialrechtlichen Versicherungsverhältnisses ausgeschlossen wird. Solange diese Frage aber nicht beantwortet ist, können rezepthafte Phagenarzneimittel allenfalls in den Ausnahmetatbeständen des § 2 Abs. 1a SGB V (bei lebensbedrohlichen, regelmäßig tödlichen, wertungsmäßig vergleichbaren Erkrankungen) erstattet werden. Zudem kommt bei Krankenhausbehandlungen eine Erstattung in Betracht, wenn das Vorliegen einer Potenzialmethode (erforderliche Behandlungsalternative) nach § 137c Abs. 3 SGB V bejaht wird, wobei auch dies nur für die Behandlung schwerwiegender Erkrankungen einschlägig ist, für die im Einzelfall keine andere Standardbehandlung verfügbar ist [35]. Für die Translation in die Regelversorgung sind diese Ausnahmen jedoch keine Basis.

## Regulatorischer Ausblick

Mit dem im April 2023 von der EU-Kommission vorgeschlagenen EU-Pharmapaket soll u. a. die RL 2001/83/EG, die Grundlage des EU-weit harmonisierten Arzneimittelrechts, durch eine neue Richtlinie ersetzt werden [36]. Im Kommissionsentwurf und im vom Europäischen Parlament im April 2024 angenommenen Änderungstext [37] zur neuen Richtlinie werden zwar für phagenbasierte Arzneimittel spezifische Vorschriften vorgeschlagen. Allerdings ist der Vorschlag nicht geeignet, patientenindividuelle Phagentherapeutika in der EU rechtlich zu harmonisieren. Die vom Reformvorschlag gemäß Art. 28 i. V. m. Anhang VII explizit für Phagenarzneimittel, die eine unterschiedliche Zusammensetzung aufweisen, geforderten angepassten Rahmenbedingungen sind aus heutiger Sicht weitestgehend ohne rechtliche Anwendungsrelevanz. Das geht zum einen darauf zurück, dass der Reformvorschlag – wie auch schon die heutige Richtlinie – nicht für patientenindividuell und damit typischerweise variabel zusammengesetzte und in Apotheken hergestellte Phagentherapeutika gelten wird, da auch in Zukunft in Apotheken hergestellte Arzneimittel nach Art. 1 Abs. 5 lit. a) des Vorschlags (heute Art. 3 Nr. 1 RL 2001/83/EG) nicht unter das EU-Recht fallen würden. Diese Arzneimittel werden daher jeweils mitgliedstaatlich reguliert und nicht durch die im Reformvorschlag geforderten angepassten Rahmenbedingungen der EU. Da die im Reformvorschlag geforderten Bestimmungen zudem nur für Phagenarzneimittel mit unterschiedlicher Zusammensetzung gelten sollen, wären selbst die im Voraus patientenunabhängig hergestellten Phagenarzneimittel mit gleichbleibender Zusammensetzung, also insbesondere die Phagencocktails, nicht erfasst. Es bleibt daher zu klären, was der tatsächliche Anwendungsbereich dieses Reformvorschlags sein soll.

Die gegenwärtig in Erstellung befindliche S2k-Leitlinie zur personalisierten Phagentherapie [38] betrifft Fragen der ärztlichen Diagnostik und Therapie, hat aber keinen Einfluss auf die arzneimitterechtliche Klassifikation, Herstellung oder Zulassung von Phagenarzneimitteln. Eine andere Frage ist, inwieweit die Leitlinie den medizinischen Standard zur Phagenanwendung, die bislang nur als Heilversuch erfolgt, verschiebt.

## Fazit

Das Arzneimittelrecht enthält kein Verbot von Phagenarzneimitteln, es handelt sich auch nicht um bedenkliche Arzneimittel i. S. v. § 5 AMG, deren Anwendung verboten wäre. Auch die fehlende Zulassung bei rezepthafter Herstellung steht einer Anwendung nicht entgegen, da es kein entsprechendes Verbot gibt. Stattdessen gibt das Arzneimittelrecht auch für rezepthafte Phagenarzneimittel den Rahmen für die Verkehrsfähigkeit dieser Arzneimittel vor. Ob diese arzneimittelrechtlichen Vorgaben in Anbetracht der Chancen und Risiken für die gegenwärtigen und die in Entwicklung befindlichen Phagenarzneimittel angemessen sind, muss der künftige interdisziplinäre Diskurs klären.

## Footnotes

1 Eine abweichende Ansicht wird im Artikel von Fürst-Wilmes et al. in diesem Themenheft dargestellt.

2 S. dazu den Beitrag von Zillmann in diesem Themenheft.

3 Bei Manuskripterstellung ist beim BVerwG zur Klärung ein Verfahren anhängig. Vorinstanz: VGH Baden-Württemberg, Urt. v. 24.04.2024–9 S 2412/22.

4 Siehe dazu den Beitrag von Idelevich und Becker in diesem Themenheft.
